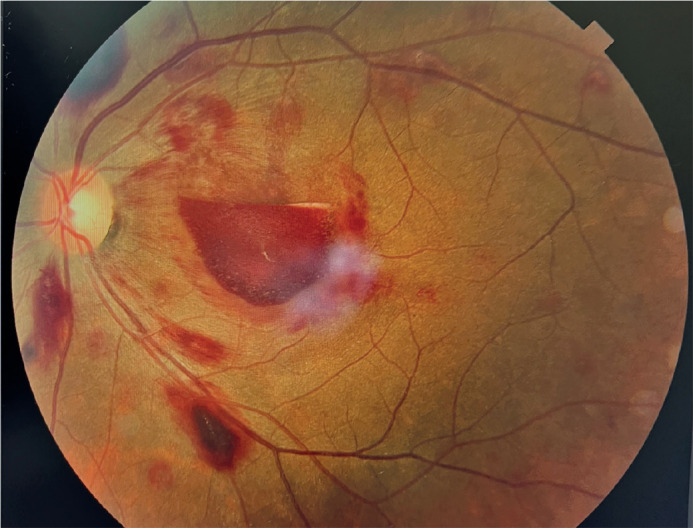# Leukemic retinopathy

**DOI:** 10.5935/0004-2749.2024-0406

**Published:** 2025-06-24

**Authors:** Carolina Minelli Martines, Nicole Bulgarão Maricondi de Almeida, Newton Kara-Junior

**Affiliations:** 1 Ophthalmology Department, Hospital das Clinicas, Universidade de São Paulo, São Paulo, SP, Brazil

Leukemia is a systemic and heterogeneous^(1)^ hematological disease, with retinal involvement as the most common ocular manifestation^(2)^. Leukemic retinopathy (Figure 1) may arise from direct infiltration or hematologic abnormalities. These manifest as intraretinal or preretinal hemorrhages and cotton-wool spots^(2)^. Treatment resolves the retinopathy within the first 2 months^(2)^. Management should be individualized based on the patients’ underlying medical condition^(1)^. If untreated, the sequelae include choroidal neovascularization and tractional retinal detachments^(2)^.

**Figure:**